# ADAMTS-13 and HMGB1-induced oxidative stress in *Taenia multiceps*-infected animals

**DOI:** 10.1038/s41598-023-44376-0

**Published:** 2023-10-20

**Authors:** Gungor Cagdas Dincel, Orhan Yavuz, Serkan Yildirim, Ebtesam M. Al-Olayan, Saeed El-Ashram

**Affiliations:** 1 Eskil Vocational School, Laboratory and Veterinary Science, Aksaray University, Aksaray, Turkey; 2 Department of Pathology, Faculty of Veterinary Medicine, Aksaray University, Aksaray, Turkey; 3 Department of Pathology, Faculty of Veterinary Medicine, Atatürk University, Erzurum, Turkey; 4 Department of Zoology, College of Science, King Saud University, 11451 Riyadh, Saudi Arabia; 5 Zoology Department, Faculty of Science, Kafrelsheikh University, Kafr El-Sheikh, 33516 Egypt; 6 College of Life Science and Engineering, Foshan University, 18 Jiangwan Street, Foshan, 528231 Guangdong Province China

**Keywords:** Immunology, Microbiology, Zoology

## Abstract

This study investigated the cytotoxic effects of oxidative stress (OS), high mobility group box 1 (HMGB1), ADAMTS (A disintegrin and metalloproteinase with thrombospondin motifs), and neuropathology associated with coenurus cerebralis (*Taenia multiceps*). ADAMTS-13, HMGB1, glutathione reductase (GR), copper/zinc superoxide dismutase (Cu/Zn SOD), and 8-hydroxy-2'-deoxyguanosine (8-OHdG) expression levels were studied. The study found that ADAMTS-13 (*P* < 0.005), HMGB1 (*P* < 0.005), GR (*P* < 0.005), Cu/Zn SOD (*P* < 0.005), and 8-OHdG (*P* < 0.005) levels were significantly higher in *T. multiceps* (c. cerebralis)-infected animals compared to healthy control animals. This study's most important finding was that HMGB1 up-regulation in neurons, endothelial cells, and glial cells can directly cause brain parenchymal destruction and that HMGB1-mediated oxidative stress plays a crucial role in the neuropathogenesis of coenurosis. The results also showed that increased levels of ADAMTS-13 may play a pivotal role in regulating and protecting the blood–brain barrier integrity and neuroprotection. These findings also suggest that ADAMTS-13 and HMGB1 compete in the prevention or formation of microthrombi, which was regarded as a remarkable finding. ADAMTS-13 and HMGB1 are valuable biomarkers for disease risk assessment, estimating host neuropathy following *T. multiceps* (c. cerebralis) exposure, and providing a new therapeutic target. This is the first study to show that HMGB1 and ADAMTS-13 are expressed in reactive cells and are associated with neuroimmunopathology in coenurosis.

## Introduction

Coenurosis is a zoonotic parasitic disease that is typically seen in small ruminants. It is caused by coenurus cerebralis, the larval stage of *Taenia multiceps*. The parasite's final hosts are carnivores such as dogs, coyotes, and foxes^1,2^. This parasitic disease is significant because it can be fatal for sheep and goats and cause life-threatening human complications^3,4^. Clinical signs in naturally and experimentally infected sheep and goats include ataxia, incoordination, lethargy, paralysis, moving in a circle, seizures, and blindness^5,6^.

High mobility group box 1 (HMGB1) is a widely expressed and highly abundant protein that has been shown to play many roles in the pathogenesis of inflammation-related diseases^7,8^. The important point here is its close relationship with neuropathology. HMGB1 is pro-inflammatory in neurodegenerative diseases such as Alzheimer's disease^9,10^. However, it has been revealed that HMGB1 exacerbates inflammation, paracrine and autocrine after ischemic brain injury and increases cytokine activity by being released from necrotic neurons and glial cells^11–13^. In addition, increased HMGB1 levels are a remarkable finding, especially in patients with major inflammatory diseases such as meningitis^14–16^. It has been demonstrated that endothelial damage occurs due to increased HMGB1 expression in diabetic neuropathy and contributes significantly to brain damage^17^. Most importantly, HMGB1 is involved in cognitive impairment^18^. These studies show that HMGB1 expressions play an essential role in the neuropathogenesis of diseases and contribute to the worsening of neuropathology. HMGB1 may damage the blood–brain barrier (BBB).

HMGB1 has been found to enhance BBB permeability and damage brain endothelial cells and pericytes^19^. In neurological diseases such as cerebral ischemic stroke, elevated HMGB1 levels exacerbate BBB damage^20,21^. Diabetic neuropathy increases BBB permeability owing to HMGB1 levels^17,18^.

A disintegrin and metalloprotease with thrombospondin type I repeats 13 (ADAMTS-13) are mainly produced by hepatic stellate cells in the liver^22^. However, recent research has revealed that it is also expressed in endothelial cells, glial cells, and neurons and plays a crucial role in neuroprotection^23–25^. ADAMTS-13 has been described as protective in neuroparenchymal tissue in ischemia–reperfusion brain injury^26^. In mice with subarachnoid hemorrhage, recombinant ADAMTS-13 treatments have shown significant reductions in microthrombus areas and damaged brain tissue^27^. In addition, it has been revealed in experimental stroke models that ADAMTS-13 deficiency exacerbates neuronal damage and contributes to the recovery process by reducing neuropathology following treatment with ADAMTS-13^28,29^. It has been reported that von Willebrand Factor, closely related to ADAMTS-13, has important roles in BBB permeability^30^. In addition, it has been emphasized that ADAMTS-13 undertakes neuroprotective functions in revealing the BBB damage in the brains of small ruminants infected with *Pestivirus*es^25^ and in listeria encephalitis^31^, and by mitigating this damage. Likewise, BBB damage in toxoplasmic encephalitis^24^ and diabetic encephalopathy^32^ has been clearly demonstrated by ADAMTS-13 expressions, and its neuroprotective functions have been explained. These studies show that ADAMTS-13 has important roles in the BBB and neuronal parenchyma, revealing the severity of BBB damage and alleviating neuropathology.

Oxidative stress (OS) occurs when there is an imbalance between pro-oxidant and anti-oxidant mechanisms such as Glutathione reductase (GR), Copper-zinc superoxide dismutase (Cu/Zn SOD)^33,34^. All cellular molecules, such as lipids, proteins and especially nucleic acids, are highly susceptible to oxidative damage^35,36^. OS has been shown to induce BBB damage, responsible for the disruption of tight junction proteins in the BBB, and increased BBB permeability with altered blood flow^37,38^. As a result, OS and damaged BBB cause neurotoxicity and eventually irreversible neuropathology^37,39^. Neuronal cells are seen to be more sensitive to oxidative damage than other tissue cells^36^. OS is also very effective in triggering neuronal dysfunction and neurodegenerations and is one of the main causes of neuronal damage in many neurodegenerative diseases^40^.

There needs to be a comprehensive study investigating the molecular pathogenesis of Coenurus cerebralis-related neuropathology. This study aimed to determine whether there is a correlation between the previously unexplored HMGB1 expressions in *T. multiceps* (c. cerebralis)-infected sheep and lamb brains and the neuropathology caused by oxidative stress. In addition, the focus was on revealing the neuropathological severity of the disease and whether there is BBB damage with ADAMTS-13 expressions, which have never been investigated before. Therefore, this study aimed to investigate the molecular mechanism of neuroimmunopathogenesis associated with Coenurus cerebralis.

## Results

### Parasitologic results

Clusters of rosebush protoscolex typical of *Taenia multiceps* were visible with no magnification or special staining. The number of scolexes per cyst ranged from 23 in the small to approximately 164 in the larger one.

### Clinicopathologic findings

It was found that cyst volumes and clinicopathological findings in *T. multiceps* (c. cerebralis)**-**infected lambs and sheep had a very close relationship. The clinicopathological results became more severe as cyst volumes and numbers increased. The most common neurological symptoms observed in *T. multiceps* (c. cerebralis)**-**infected animals were lateral deviation of the head and circling motion around its axis, paralysis, incoordination, and ataxia. Other findings included nystagmus, torticollis, a lack of direct light reflex in the pupil, seizures, and coma.

### Gross findings

**Figure 1:**
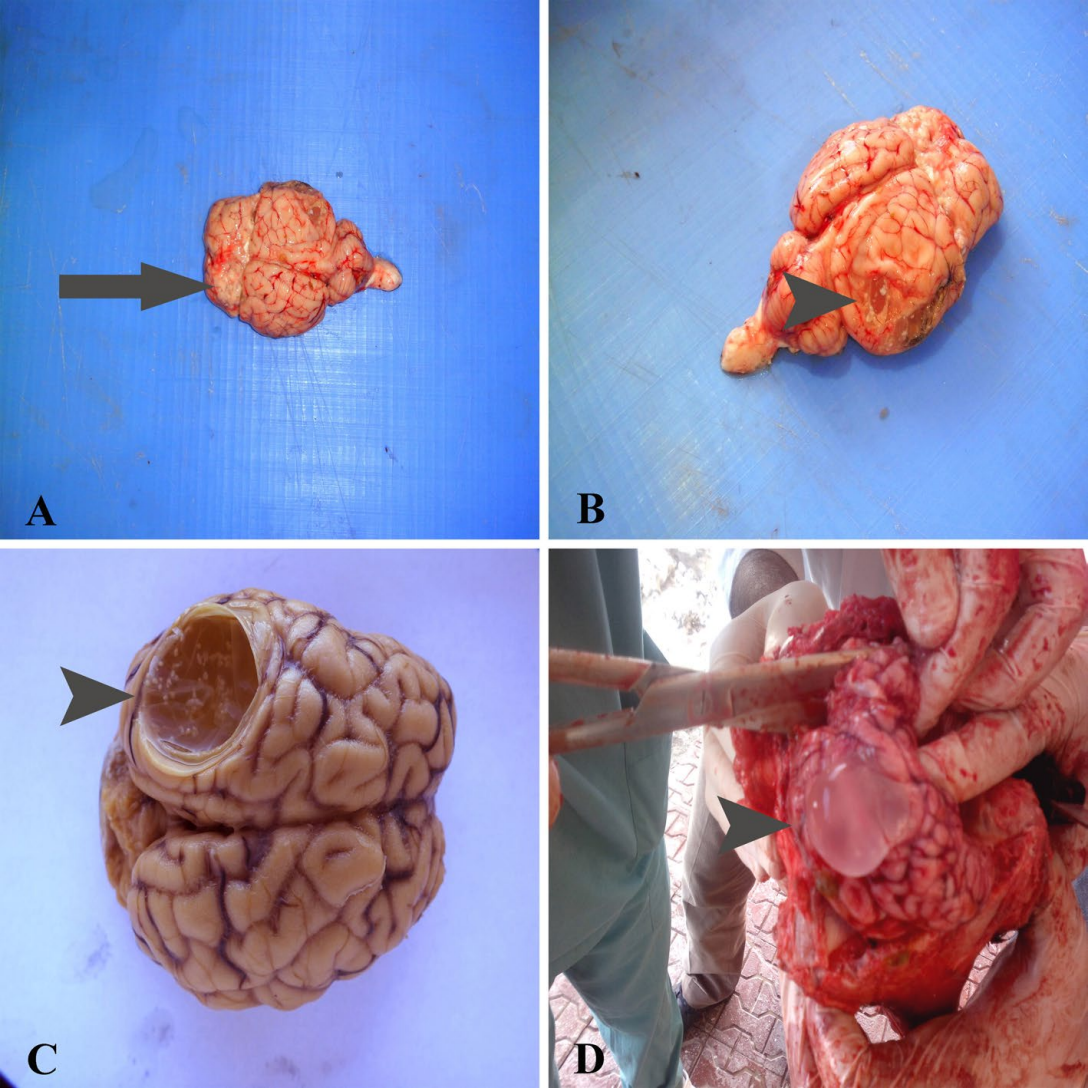
Gross pathology findings of *T. multiceps* (c. cerebralis)-infected animals. (**A**) *C. cerebralis* cyst and non-purulent meningoencephalitis area (arrow). (**B**) *C. cerebralis* cyst, scolexes and atrophy of surrounding tissue (arrowhead). (**C**) Cerebral hemisphere region showing cystic spaces due to *c. cerebralis* causing atrophy of brain tissue (arrowhead). (**D**) *C. cerebralis* cyst and scolexes (arrowhead).

Macroscopically, white-colored protoscolex bundles ranging from 23 to 164 were found within the thin-walled, transparent, and clear fluid-filled cysts (Fig. 1B–D). The collected cysts were observed to be 2.4 × 3.3 cm–5.8 × 7.6 cm in diameter and varied in size from a ping-pong ball to a baseball (Fig. 1C,B). Sometimes, a sizeable unilateral cyst was found; in others, two to three small or large cysts were observed in different locations (Table 1). In the evaluation made according to the cyst locations in the brain, there was a more widespread distribution predominantly in the right or left cerebral hemispheres. Cysts were found in the cerebellum in only three cases (16.6%) and in the thalamus in one case (5.5%). When the cyst locations in the cerebral hemispheres were examined, it was seen that the cysts were in the right or left parietal, frontal, temporal and occipital lobes (Table 1). Because of the pressure atrophy caused by the cysts occupying the brain parenchyma (Fig. 1A–C), it was observed that the cyst area became thinner and even the skull's inner surface softened in all cases.

**Table 1:** Cyst location, dimensions and scolex numbers.

No	Sex	Age	Number of cysts	Location of cysts	Diameter of cyst (cm)	Scolex number
Lamb 1	Female	8 months	1	Right temporal lobe	4.1 × 5.9	74
Lamb 2	Female	9 months	1	Left parietal lobe	5.3 × 6.6	147
Lamb 3	Female	9 months	2	Left occipital lobe	4.2 × 5.3	92
				Left parietal lobe	3.7 × 4.2	
Lamb 4	Male	8 months	2	Right temporal lobe Left parietal lobe	2.4 × 3.3	23
Lamb 5	Male	8 months	2	Left temporal lobe	2.9 × 3.7	69
				Cerebellum	3.1 × 3.8	
Lamb 6	Male	9 months	1	Right temporal lobe	2.4 × 3.6	50
Lamb 7	Male	10 months	3	Thalamus	2.9 × 3.7	157
				Right parietal lobe (2)	4.3 × 4.9–5.1 × 5.5	
Lamb 8	Male	10 months	1	Left occipital lobe	4.4 × 5.1	82
Sheep 1	Female	17 months	2	Right temporal lobe	2.8 × 3.3	98
				Left parietal lobe	4.7 × 5.1	
Sheep 2	Female	20 months	1	Left temporal lobe	6.4 × 6.9	151
Sheep 3	Female	25 months	3	Right temporal lobe	3 × 4	164
				Left parietal lobe	2.9 × 3.1	
				Right parietal lobe	4.3 × 5.6	
Sheep 4	Female	28 months	2	Right Frontal Lobe	5.8 × 7.6	131
				Left parietal lobe	3.1 × 4.7	
Sheep 5	Male	22 months	1	Left parietal lobe	2.6 × 3.4	77
Sheep 6	Male	26 months	1	Left temporal lobe	5 × 5.6	90
Sheep 7	Male	28 months	2	Right frontal lobe Cerebellum	4.8 × 5.3	119
Sheep 8	Male	29 months	2	Right temporal lobe	2.9 × 3	156
				Left parietal lobe	3.4 × 4.1	
Sheep 9	Male	29 months	2	Right occipital Lobe	2.5 × 2.8	148
				Cerebellum	4.2 × 4.6	
Sheep 10	Male	32 months	1	Left parietal lobe	5.2 × 5.5	87

### Histopathologic findings

**Figure 2:**
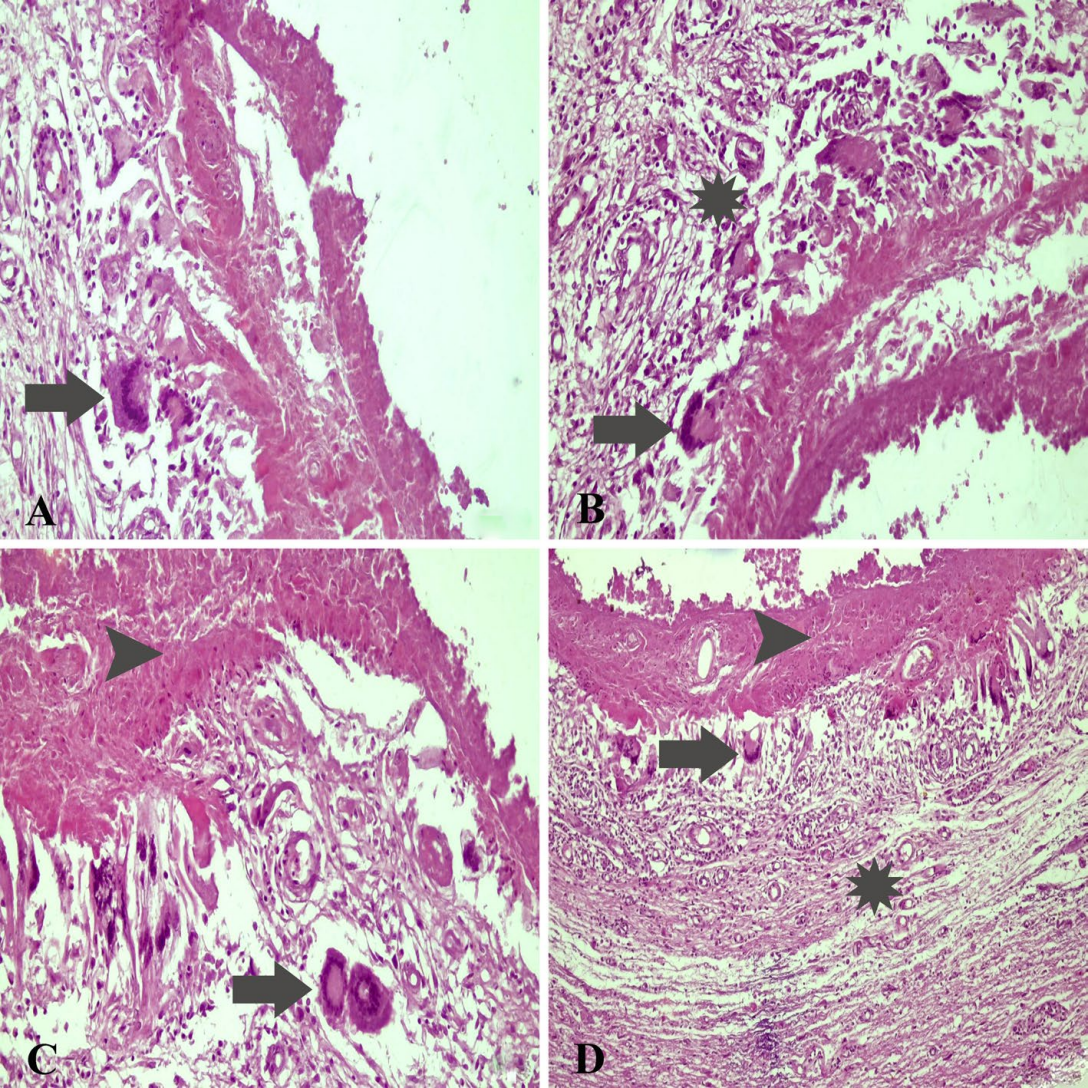
Histopathological findings of *T. multiceps* (c. cerebralis)-infected animals. (**A**) Non-purulent meningoencephalitis, areas of necrosis around the cyst membrane and numerous foreign body giant cells (arrow) against the cyst wall. (**B**) Foreign body giant cells (arrow) against the cyst wall and inflammatory zone area (mononuclear cell infiltration) (asterisk). (**C**) Numerous foreign body giant cells (arrow) and extensive areas of necrosis (arrowhead) (**D**) Extensive areas of necrosis (arrowhead), numerous foreign body giant cells (arrow) against the cyst wall and large inflammatory zone area (mononuclear cell infiltration) (asterisk).

Hematoxylin and eosin-stained brain sections from healthy control animals exhibited standard architecture. Significant histopathological lesions were observed in the brain tissues of all animals. Histopathological examination revealed many multinucleated giant cells surrounding the cyst walls and lymphocytic reactions surrounded by inflammatory cells (Fig. 2A–D). Severe hyperemia and edema were detected in the meninges of *T. multiceps* (c. cerebralis)-infected animals. In addition, demyelination, liquefaction necrosis around cerebral cysts, degeneration of neurons, focal pressure atrophy, perivascular cell infiltrates, thrombus and fibrinopurulent meningoencephalitis were noted as the most important histopathological findings (Fig. 2A–D).

### Immunoperoxidase findings

**Figure 3:**
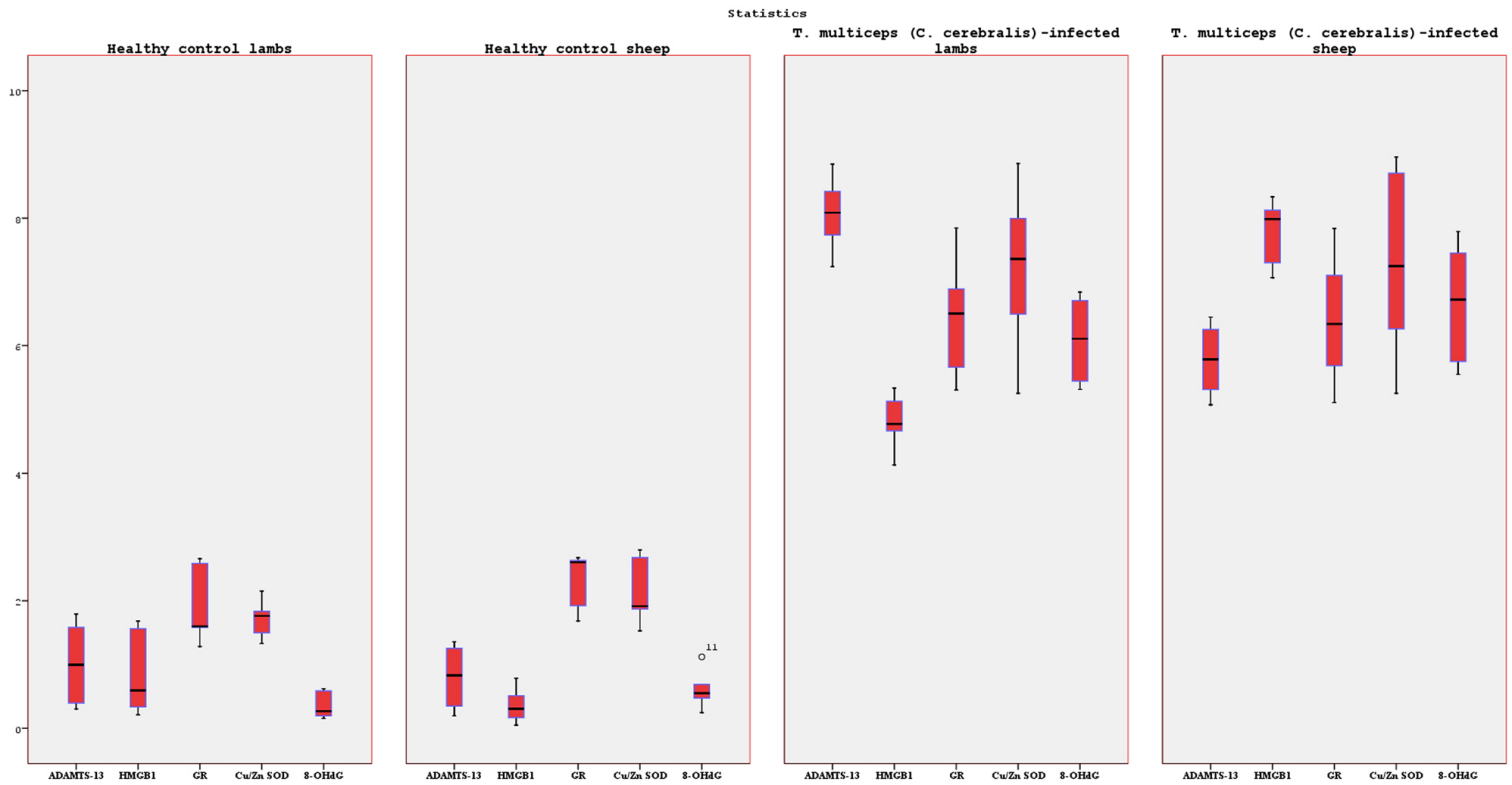
Significant up-regulation of ADAMTS-13, HMGB1, GR, Cu/Zn SOD and 8-OHdG expressions in brains in *T. multiceps* (c. cerebralis)-infected animals.

We analyzed the expression levels of ADAMTS-13, HMGB1, GR, Cu/Zn SOD, and 8-OHdG in the brain tissues from *T. multiceps* (c. cerebralis)-infected and healthy control animals. Immunohistochemical analysis showed significant up-regulation of ADAMTS-13, HMGB1, GR, Cu/Zn SOD, and 8-OHdG expressions in the *T. multiceps* (c. cerebralis)-infected lambs and sheep, unlike that in the case of the healthy control animals. Statistical data analysis on ADAMTS-13, HMGB1, GR, Cu/Zn SOD, and 8-OHdG expressions in the brain, measured by immunostaining in all the groups, are listed in Fig. 3 and Tables 2, 3, 4, 5. According to the comparison between *T. multiceps* (c. cerebralis)-infected lambs and sheep, ADAMTS-13 expressions were found to be statistically significantly higher in *T. multiceps* (c. cerebralis)-infected lambs than in *T. multiceps* (c. cerebralis)-infected sheep (*P* < 0.001) (Table 4). Conversely, HMGB1 expressions of sheep infected with *T. multiceps* (c. cerebralis) were statistically higher than lambs infected with *T. multiceps* (c. cerebralis) (*P* < 0.001) (Table 4). Statistically significant differences were not observed in GR, Cu/Zn SOD and 8-OHdG expressions between lambs and sheep infected with *T. multiceps* (c. cerebralis) (Table 5).

**Table 2:** Immunoperoxidase test results and statistical data for healthy control and *T. multiceps* (c. cerebralis)-infected animals.

Animals	n	ADAMTS-13		Between-component variance ADAMTS-13	*P*	HMGB1		Between-component variance HMGB1	*P*
		Mean	SD			Mean	SD		
Healthy control lambs	6	1.011	0.635			0.8293	0.639		
					0.002				0.002
*T. multiceps* (c. cerebralis)*-*infected lambs	8	8.071	0.533			4.825	0.378		
				12.313				13.111	
Healthy control sheep	6	0.802	0.493			0.353	0.266		
					0.002				0.002
*T. multiceps* (c. cerebralis)*-*infected sheep	10	5.781	0.51			7.817	0.461

**Table 3:** Immunoperoxidase test results and statistical data for healthy control and *T. multiceps* (c. cerebralis)-infected animals.

Animals	n	GR		Between-component variance GR	*P*	Cu/Zn SOD		Between-component variance Cu/Zn SOD	*P*	8-OHdG		Between-component variance 8-OHdG	*P*
		Mean	SD			Mean	SD			Mean	SD		
Healthy control lambs	6	1.885	0.585			1.723	0.285			0.348	0.202		
					0.002				0.002				0.002
*T. multiceps* (c. cerebralis)-infected lambs	8	6.407	0.835			7.225	1.128			6.082	0.635		
				5.871				9.102				11.518	
Healthy control sheep	6	2.355	0.434			2.117	0.503			0.604	0.294		
					0.002				0.002				0.002
*T. multiceps* (c. cerebralis)-infected sheep	10	6.368	0.906			7.26	1.343			6.687	0.822

**Table 4:** Immunoperoxidase test results and statistical data for *T. multiceps* (C. cerebralis)-infected animals.

Animals	n	ADAMTS-13		*P*	HMGB1		*P*
		Mean	SD		Mean	SD	
*T. multiceps* (c. cerebralis)-infected lambs	8	8.071	0.533	0.000	4.825	0.378	0.000
*T. multiceps* (c. cerebralis)-infected sheep	10	5.781	0.51		7.817	0.461

**Table 5:** Immunoperoxidase test results and statistical data for *T. multiceps* (c. cerebralis)-infected animals.

Animals	n	GR		*P*	Cu/Zn SOD		*P*	8-OHdG		*P*
		Mean	SD		Mean	SD		Mean	SD	
*T. multiceps* (c. cerebralis)-infected lambs	8	6.407	0.835	0.929	7.225	1.128	0.929	6.082	0.635	0.091
*T. multiceps* (c. cerebralis)-infected sheep	10	6.368	0. 906		7.26	1.343		6.687	0.822	

### ADAMTS-13 and HMGB1 expressions

**Figure 4:**
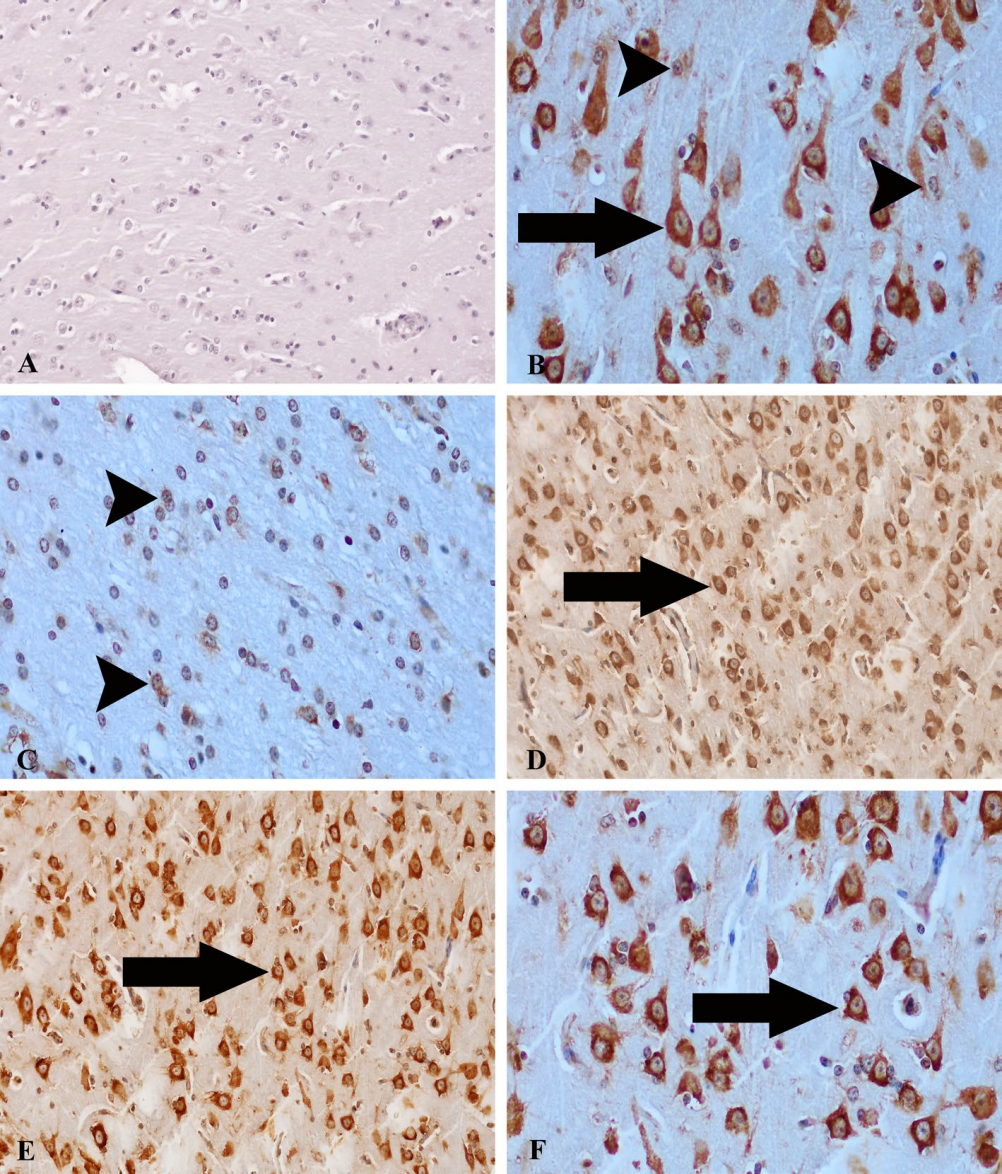
ADAMTS-13 expressions in healthy control and *T. multiceps* (c. cerebralis)-infected animals. (**A**) Weak ADAMTS-13 expression in glial, endothelial cells and neurons of healthy control animals. ABC technique (anti-ADAMTS-13), Mayer's hematoxylin counterstain. (**B**) Strong expression of ADAMTS-13 in glial (arrowheads) and neuronal cells (arrow). ABC technique (anti-ADAMTS-13), Mayer's hematoxylin counterstain. (**C**) Strong expression of ADAMTS-13 in glial cells (arrowheads). ABC technique (anti-ADAMTS-13), Mayer's hematoxylin counterstain. (**D**,**E**,**F**) Strong expression of ADAMTS-13 in neuronal cells (arrows). ABC technique (anti-ADAMTS-13), Mayer's hematoxylin counterstain.

**Figure 5:**
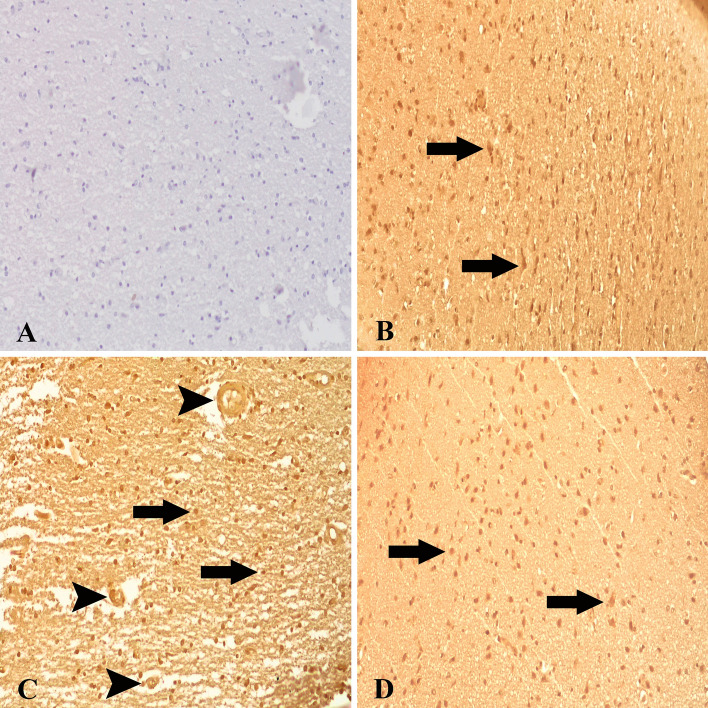
HMGB1 expressions in healthy control and *T. multiceps* (c. cerebralis)-infected animals. (**A**) Weak HMGB1 expression in glial, endothelial cells and neurons of healthy control animals. ABC technique (anti-HMGB1), Mayer's hematoxylin counterstain. (**B**) Strong expression of HMGB1 in healthy and necrotic neuronal cells (arrows). ABC technique (anti-HMGB1), Mayer's hematoxylin counterstain. (**C**) Strong expression of HMGB1 in glial (arrows) and endothelial cells (arrwheads). ABC technique (anti-HMGB1), Mayer's hematoxylin counterstain. (**D**) Strong expression of HMGB1 in healthy and necrotic (arrows) neuronal cells. ABC technique (anti-HMGB1), Mayer's hematoxylin counterstain.

Expressions of ADAMTS-13 and HMGB1 in all brain regions of all animals in both *T. multiceps* (c. cerebralis)*-*infected and healthy control animals were carefully measured. It was noted that ADAMTS-13 and HMGB1 expressions were increased, and the difference was statistically significant and relatively high when this increase was compared with the healthy control animals and interpreted quantitatively (*P* < 0.005) (Fig. 3 and Table 2). We detected weak ADAMTS-13 expressions in healthy control animals, glial cells and neurons (Fig. 4A). Contrary to these findings, ADAMTS-13 expressions in brain tissues of *T. multiceps* (c. cerebralis)-infected animals were significantly increased, especially in neurons (Fig. 4B–F). In addition, partial ADAMTS-13 immunoreactivities were detected in glial cells (Fig. 4C). Weak or deficient HMGB1 expressions were observed in neurons, endothelial and glial cells in healthy control animals (Fig. 5A). However, it was determined that the intensity of HMGB1 expression in the brain tissues of *T. multiceps* (c. cerebralis)-infected animals were higher than in the healthy control animals (Table 2). Pathological levels of HMGB1 expressions were found in glial and endothelial cells, especially in necrotic and healthy neurons (Fig. 5B–D). The most striking finding of this study is that the expressions of ADAMTS-13 and HMGB1 were significantly increased in the lesioned areas and the areas close to the cyst. The most striking finding of this study is that the expressions of ADAMTS-13 and HMGB1 are significantly increased in the lesioned areas and the areas close to the cyst. *T. multiceps* (c. cerebralis)-infected animal brains showed enhanced ADAMTS-13 and HMGB1 levels and prolonged OS, which may contribute to neurotoxicity and parenchymal degeneration. These conditions handle severe neuropathology, such as demyelination and thrombus and permeability of the BBB.

### Glutathione reductase expressions

**Figure 6:**
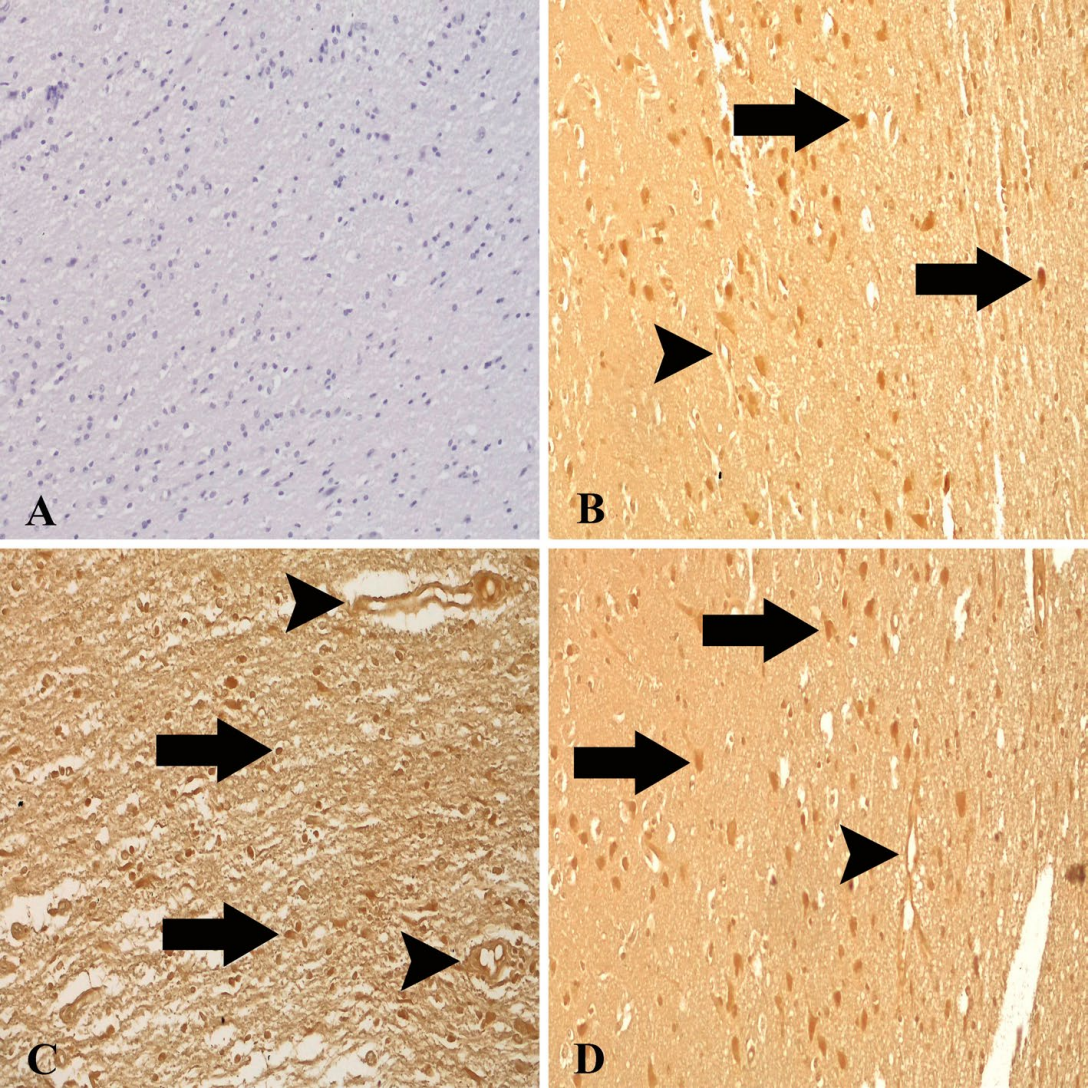
GR expressions in healthy control and *T. multiceps* (c. cerebralis)-infected animals. (**A**) Weak GR expression in glial, endothelial and neuronal cells of healthy control animals. ABC technique (anti-GR), Mayer's hematoxylin counterstain. (**B**) Strong expression of GR in neuronal (arrows) and endothelial cells (arrwhead). ABC technique (anti-GR), Mayer's hematoxylin counterstain. (**C**,**D**) Strong expression of GR in glial (arrows) and endothelial cells (arrwheads). ABC technique (anti-GR), Mayer's hematoxylin counterstain.

In healthy control animals, some glial cells and neurons exhibited relatively weak or no immunoreactivity for GR (Fig. 6A). GR expression increased significantly in the vascular endothelial, glial cells, and necrotic/healthy neurons (Fig. 6B–D), which was also substantially higher than the levels in the healthy control animals (*P* < 0.005). We detected that an important anti-oxidant marker, GR, was up-regulated considerably in *T. multiceps* (c. cerebralis)-infected animals (Fig. 3 and Table 3).

### Cu/Zn SOD expressions

**Figure 7:**
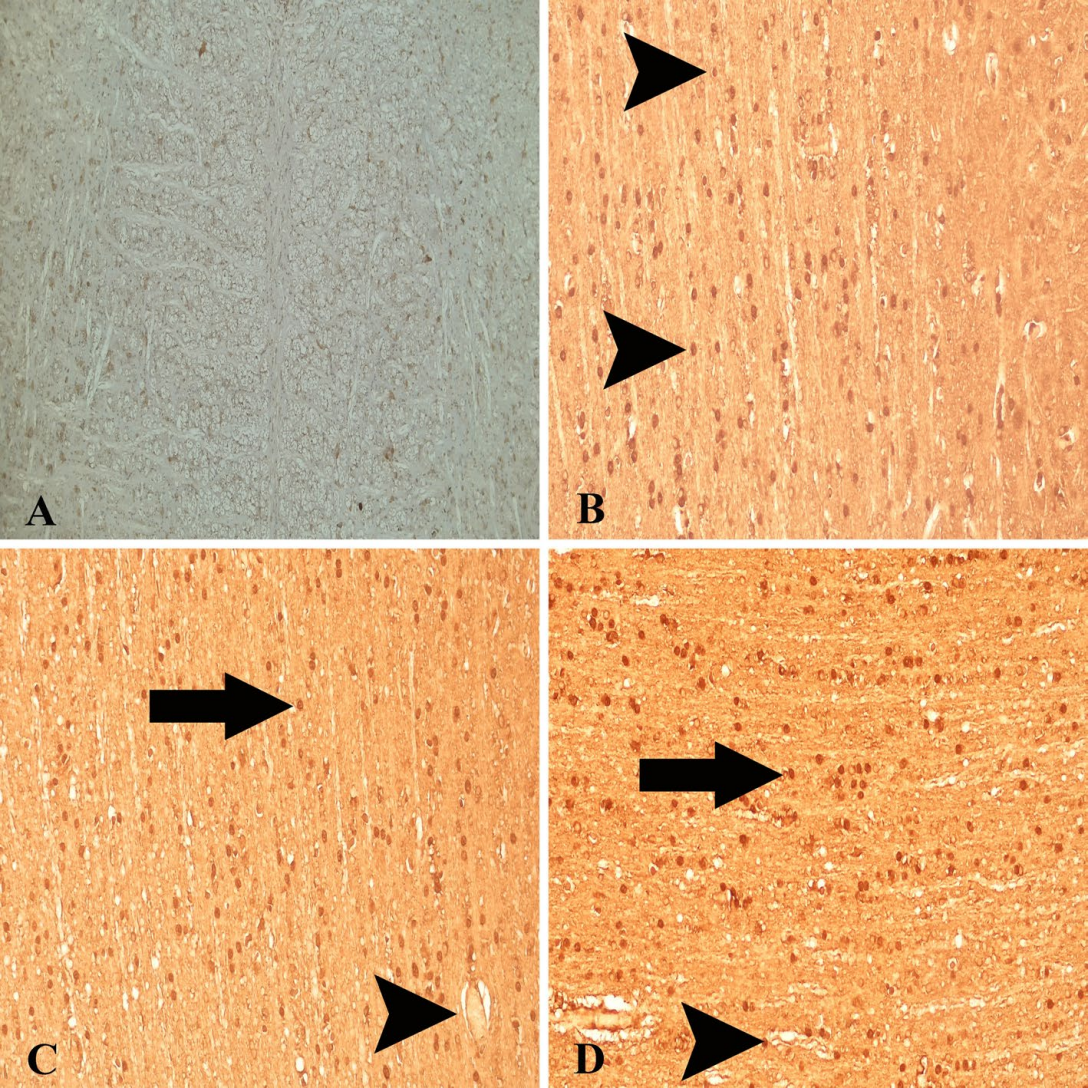
Cu/Zn SOD expressions in healthy control and *T. multiceps* (C. cerebralis)-infected animals. (**A**) Weak Cu/Zn SOD expression in glial, endothelial and neuronal cells of healthy control animals. ABC technique (anti-Cu/Zn SOD), Mayer's hematoxylin counterstain. (**B**) Strong expression of Cu/Zn SOD in glial cells (arrowheads). ABC technique (anti-Cu/Zn SOD), Mayer's hematoxylin counterstain. (**C**,**D**) Strong expression of Cu/Zn SOD in glial cells (arrows) and endothelial cells (arrowheads). ABC technique (anti-Cu/Zn SOD), Mayer's hematoxylin counterstain.

Moderate Cu/Zn SOD expressions were observed in neuronal and glial cells in healthy control animals (Fig. 7A). Another conspicuous finding of the present study was that Cu/Zn SOD expressions were markedly increased in the endothelia, glial cells and microglia/macrophages (Fig. 7B–D), which was also significantly higher than the levels in the healthy control animals (*P* < 0.005). We detected that a very important anti-oxidant marker, Cu/Zn SOD, was significantly up-regulated in *T. multiceps* (c. cerebralis)-infected animals (Fig. 3 and Table 3).

### 8-OHdG expressions

**Figure 8:**
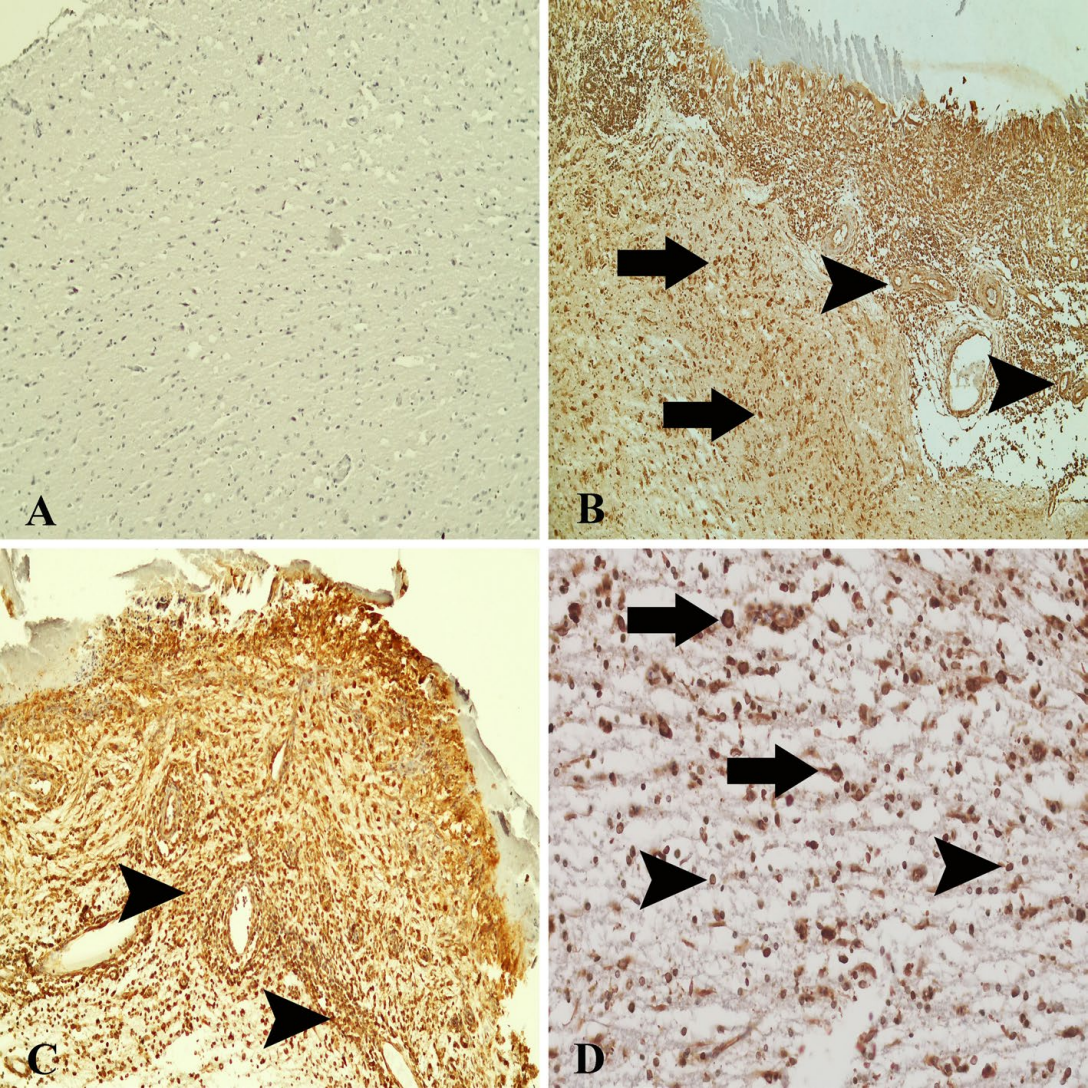
8-OHdG expressions in healthy control and *T. multiceps* (c. cerebralis)-infected animals. (**A**) Weak 8-OHdG expression in glial, endothelial and neuronal cells of healthy control animals. ABC technique (anti-8-OHdG), Mayer's hematoxylin counterstain. (**B**) Strong expression of 8-OHdG in endothelial (arrowheads), glial and neuronal cells (arrows). ABC technique (anti-8-OHdG), Mayer's hematoxylin counterstain. (**C**) Strong expression of 8-OHdG in infiltrative inflammatory cells (arrowheads), neuronal and glial cells. ABC technique (anti-8-OHdG), Mayer's hematoxylin counterstain. (**D**) Strong expression of 8-OHdG in neuronal (arrows), endothelial and glial cells (arrowheads). ABC technique (anti-8-OHdG), Mayer's hematoxylin counterstain.

In healthy control animals, some glial/neuronal cells showed weak immunoreactivity to 8-OHdG (Fig. 8A). 8-OHdG expression was observed in the nucleus and in the cytoplasm of neurons, endothelial cells, glial cells, and microglia/macrophages (Fig. 8B–D), being more intense in the cytoplasm. 8-OHdG expressions measurements in *T. multiceps* (c. cerebralis)-infected animals were significantly higher than in healthy control animals (*P* < 0.005) (Fig. 3 and Table 3). The most conspicuous finding of the present study was that 8-OHdG expression markedly increased in cyst walls.

## Discussion

**Figure 9:**
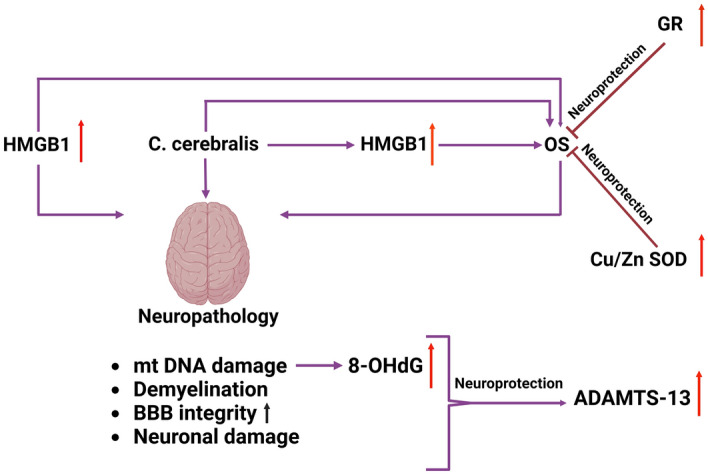
Molecular pathogenesis of neuropathology in *T. multiceps* (c. cerebralis)-infected animals.

Coenurosis is a disease caused by coenurus cerebralis, frequently seen in small ruminants, caused by the larval stage of *Taenia multiceps*. Its zoonotic character poses a severe public health problem that cannot be ignored^1,2^. There are minimal studies on the neuroimmunopathogenesis of coenurosis, a significant parasitic disease. For this reason, the findings obtained in this study are significant in elucidating the neuroimmunopathogenesis of the disease. The study's most striking first finding, a significant increase in HMGB1 expression and a HMGB1-mediated OS, has already been discussed. The second significant finding is the presence of ADAMTS-13, which is highly expressed in neurons and glial cells. It has thus been clearly demonstrated that the BBB's permeability increases, and its integrity is disrupted, contributing to the development of neuropathological findings. The third finding is that HMGB1 and ADAMTS-13 compete to form and prevent microthrombi, one of the most essential histopathological findings (Fig. 9).

There is a link between oxidative stress and HMGB1 expression^41^. OS has been shown to regulate HMGB1 translocation, release, and activity significantly^41,42^. It has been found that HMGB1 functions as a critical regulator in OS-related infection, sterile inflammation, necrosis, and apoptosis^42^. In modeling studies of neurodegeneration in which HMGB1 inhibition is performed, it has been revealed that OS and neuronal cell death are significantly reduced and that it prevents neuropathology that may occur against BBB damage^20,43,44^. These studies show that neuropathology occurs in neurodegenerative diseases because of HMGB1-mediated OS. As the most important finding of this study, we showed that HMGB1 expressed in both necrotic and healthy neurons and glial cells at pathological levels are associated with OS. It is thought that a serious increase in HMGB1 expressions is among the causes of severe mitochondrial DNA (mtDNA) damage because of OS. In short, it has been clearly demonstrated that the most crucial trigger of OS-mediated neuropathology in coenurosis is HMGB1. The significantly increased levels of HMGB1 and HMGB1-associated OS shown in this study are thought to underlie the neuropathology seen in coenurosis. The development of therapeutic agents specifically targeting HMGB1 or its receptors may help to reduce the inflammatory response and OS associated with coenurosis. Inhibition of HMGB1 release or blockade of downstream signalling pathways may contribute significantly to the reduction of neuroinflammation and related pathological processes.

The blood–brain barrier is a physical and metabolic barrier that regulates and protects the microenvironment in the central nervous system (CNS) and separates the CNS from the peripheral circulation^45^. Considering the histopathological findings, it is seen that the permeability of the BBB increases in coenurosis. For this reason, the brain parenchyma and cells become sensitive due to the increase in BBB permeability during infection and play a critical role in triggering and exacerbating inflammation. For this reason, the brain parenchyma and cells become sensitive due to the increase in BBB permeability during infection and play a critical role in triggering and exacerbating inflammation. This study emphasises two reasons for the increase in BBB permeability. 1) The first one is BBB damage occurs due to HMGB1 expression at the pathological level; 2) HMGB1-mediated OS increases BBB permeability and damages BBB integrity. Because it has been shown that increased HMGB1 expressions cause damage to brain endothelium and pericytes, disrupting BBB integrity^19,46^. Similarly, it has been reported that OS increases BBB permeability and that BBB damage caused by OS causes neurotoxicity, exacerbating neuropathology^37,38^. Another striking finding of this study was that ADAMTS-13 expression was significantly increased, particularly in neurons.

Our previous studies demonstrated that ADAMTS-13 is expressed at severe levels in endothelial cells, glial cells and neurons in *Pestivirus*-related neuropathology^25^, listeria encephalitis^31^, diabetic encephalopathy^32^ and toxoplasmic encephalitis models^24^. Thus, ADAMTS-13 indicates BBB damage severity and increased BBB permeability. Similarly, we found that the severity of the existing damage can be followed up with ADAMTS-13, and it gives essential ideas for interpreting the severity in neuropathology. In addition, we revealed that ADAMTS-13 has protective effects on the BBB. We demonstrated in this study that the significantly increased ADAMTS-13 expression in the brain tissues of sheep with coenurosis has the neuroprotective effects described above and previously revealed, protects the brain from BBB damage, and provides information about the severity of the disease. In light of the studies, the role of ADAMTS-13 in investigating Coenurus cerebralis-related neuroimmunopathogenesis is significant. This and our previous studies suggest that ADAMTS-13 may play a role in modulating vascular integrity, inflammation or coagulation processes that may affect BBB permeability. Furthermore, examination of the relationship between ADAMTS-13 and BBB damage may help to elucidate the interactions between the cells that make up the BBB and its associates.

Coenurus cerebralis has been shown histopathologically to cause demyelination in the brain^47–49^. In this study, we also found demyelination in 5 sheep. Although demyelination is a significant neuropathology, no studies have been conducted to investigate the pathogenesis of demyelination in this disease. We previously investigated the role of ADAMTS-13 in preventing hydranencephaly, cerebellar hypoplasia, and, in particular, hypomyelinogenesis in *Pestivirus*-infected sheep. Furthermore, we discussed in the same study that it protects against the formation of myelin damage. The increase in ADAMTS-13 expression observed in this study may also play a role in demyelination prevention. It is obvious that when ADAMTS-13 expression levels are high enough, myelin damage will stop. Although demyelination is seen, the fact that it was not found in all sheep with coenurosis brains supports our theory.

The roles of ADAMTS-13 in thrombotic activity are significant. It has been revealed to prevent further deterioration of disease prognosis by cleaving ultra-large von Willebrand factor multimers into multimers with lower activity to reduce potential thrombotic activity and prevent microvascular thrombosis^50,51^. Mice with subarachnoid hemorrhage have shown statistically significant reductions in microthrombus areas and attenuated brain tissue damage after treatment with recombinant ADAMTS-13^27^. In contrast to ADAMTS-13, HMGB1 has been shown in an animal model of deep vein thrombosis to induce pro-thrombotic neutrophil extracellular trap formation and in vivo/in vitro platelet aggregation^52–54^. These studies show that increased HMGB1 activations at pathological levels may expose patients to a potential stroke. Thrombosis in coenurosis has also been linked to severe neuropathology^55,56^. This indicates that disturbances in thrombotic activity in coenurosis play an essential role in neuropathogenesis. At this time, it is believed that ADAMTS-13 and HMGB1 have a competitive interaction. Demyelination would not be interpreted this way if it were considered a universal neurohistopathological finding in all animals. However, the absence of demyelination in animals with coenurosis leads us to believe that ADAMTS-13 and HMGB1 compete as winners and losers. With HMGB1 promoting thrombus formation and ADAMTS-13 counteracting it, it is quite clear that an imbalance in favour of HMGB1 could lead to increased microthrombus formation and potentially contribute to tissue damage in coenurosis-related neuropathology. This demonstrated important finding suggests that targeting HMGB1 to reduce its pro-inflammatory and pro-thrombotic effects or increasing ADAMTS-13 activity to reduce microthrombus formation could be considered as potential interventions to limit disease progression.

In conclusion, this study revealed previously unknown neuroimmunopathogenesis of neuropathology in sheep with coenurosis. Damage to the BBB and increased permeability are visible. It is seen that HMGB1 alone and HMGB1-mediated OS are responsible for the damage and increased permeability of this BBB. In addition, the presence of mtDNA damage caused by OS was demonstrated by 8-OHdG expressions. In addition, it is thought that demyelination, an important neuropathological finding, is prevented with ADAMTS-13 expression. In addition, it was seen as a remarkable finding that ADAMTS-13 and HMGB1 play a competitive role in the prevention or formation of microthrombi.

Understanding the mechanisms underlying neuronal injury and inflammation in coenurosis provides important information for the development of neuroprotective interventions. With the findings obtained in this study, strategies that reduce neuronal plasticity and oxidative stress or promote tissue repair mechanisms can be investigated as potential therapeutic avenues. For example, targeting these factors, such as specific parasite proteins or antigens, may be among the first targets to reduce disease severity and promote recovery.

## Materials and methods

### Ethics statement

Aksaray and Atatürk University's Animal Experiments Ethics Committee did not require approval. Because this study did not include any invasive procedures for animal experiments. The study samples comprised 18 dead lambs and sheep, ranging in age from 8 to 36 months. These animals were brought to Aksaray and Atatürk University, Faculty of Veterinary Medicine, Department of Pathology for routine necropsy. No animals studied were sacrificed for this study, and all procedures were performed with the permission of the animal owners. As a healthy control animal, a total of 6 lambs and 6 sheep brains slaughtered for human consumption from Erzurum Slaughterhouse were used. Only the heads of these healthy animals sacrificed in the slaughterhouse were purchased.

### Parasitological examinations

When the skulls of 18 lambs and sheep were opened, fluid-filled cysts in different regions were encountered. The cysts were carefully removed without rupture and placed in Petri dishes containing 70% ethyl alcohol. The structure and content of each cyst were examined both macroscopically and microscopically, and the advanced scolex structures were investigated. The gross and morphological appearance of cysts, their dimensions and their location in the brain were recorded and photographed.

### Pathologic examination

The healthy control animals compromised the brain samples of slaughtered healthy lambs (n = 6) and sheep (n = 6). No findings were found in the brain tissues of these animals, both macroscopically and histopathologically. All steps followed the procedure described by Dincel and Kul^57^. The brains were removed and fixed in 4% paraformaldehyde in phosphate-buffered saline (PBS) at pH 7.4 for 48 h and then were thoroughly rinsed overnight, under tap water. After performing the routine tissue preparation procedures of dehydration using graded alcohol and xylene, the tissue samples were embedded in paraffin blocks; 4–5 μm thick paraffin sections were then cut and mounted on glass slides. Hematoyxlin-Eosin (H&E) and immunohistochemical tests were performed, and they were analyzed using a trinocular light microscope (Olympus BX51 and DP25 digital camera). The severity of coenurus cerebralis in each animal was classified according to neuropathological changes: demyelination, hyperemia, mononuclear cell infiltrates, giant cells around the cystic membrane, gliosis, astrocytosis, neuronal degeneration and malaise.

### Antibodies

Commercial antibodies against ADAMTS-13 (Abcam, Cambridge, UK) diluted to 1:100, HMGB1 (Thermo Scientific, USA) diluted to 1:200, GR (Santa Cruz Biotechnology Dallas, TX, USA) diluted to 1:250, Cu/Zn SOD (Santa Cruz Biotechnology Dallas, TX, USA) diluted to 1:400, and 8-hydroxy-2-deoxyguanosine (8-OHdG) (Santa Cruz Biotechnology, Dallas, TX, USA) diluted to 1:500, were used.

### Immunoperoxidase examinations

Immunohistochemistry was performed to observe ADAMTS-13, HMGB1, GR, Cu/Zn SOD and 8-OHdG in the 4–5 μm-thick paraffin sections of the tissues by using an indirect streptavidin/biotin immunoperoxidase kit (HRP, Thermo Scientific, USA), as per the manufacturer's instructions. All steps followed the procedure described by Dincel and Kul^57^. Briefly, the sections were placed onto adhesive slides and deparaffinized for 5 min. Each was in the 3-step xylene series, and rehydrated using a series of graded alcohol and distilled water. The antigens were retrieved by boiling the tissue sections on glass slides in citrate buffer (pH 6.0) (Thermo Scientific, USA) for 20 min. Endogenous peroxidase activity was quenched using 3% hydrogen peroxide in absolute methanol for 7 min at room temperature (RT). The tissue sections were rinsed thrice with phosphate buffer solution (pH 7.4) for 5 min, between each consecutive step. The sections were then incubated in a blocking serum for 5 min to prevent non-specific antibody binding. The sections were incubated with ADAMTS-13, HMGB1, GR, Cu/Zn SOD and 8-OHdG antibodies for 60 min in a humidity chamber at the RT. After treating the sections with biotinlabeled secondary antibody for 15 min and streptavidin-peroxidase enzyme for 15 min at RT, the colour reaction was performed using 3,3'-Diaminobenzidine chromogen for 5–10 min. Sections were counterstained with Mayer's hematoxylin for 1–2 min and suspended in water-based mounting medium (Thermo Scientific, USA). For each immunoperoxidase test, six (3 lambs and 3 sheep) negative control tissue sections were allowed as follows; as a negative control, one of the serial paraffin sections was incubated with normal mouse serum (isotype serum control) instead of primary antibody. The primary antibody step was also omitted to control non-specific endogenous peroxidase and biotin activities in each test.

### Histomorphometric analysis and statistics

The density of positive staining was measured using a computerized image system composed of a Leica CCD camera DFC420 (Leica Microsystems Imaging Solutions, Ltd., Cambridge, UK) connected to a Leica DM4000 B microscope (Leica Microsystems Imaging Solutions, Ltd.). Five representative fields were selected, and consecutive pictures were captured by Leica QWin Plus v3 software under 20 × objective lens (Leica Microsystems Imaging Solutions, N Plan) at a setting identical to the imaging system for analysing. We used the same setting for all slides. The integrated optical density of all ADAMTS-13-, HMGB1-, GR-, Cu/Zn SOD-, and 8-OHdG-positive staining were measured, and the mean ADAMTS-13-, HMGB1-, GR-, Cu/Zn SOD-, and 8-OHdG-positive area/total area was calculated by Leica QWin Plus v3. All images were collected under the same lighting conditions. To avoid observer bias, a blinded investigator quantified all sections. Data were described in terms of mean and standard deviation (mean ± SD) for area %. After calculating the proportion (% pixels) of the stained area to the whole field, each slide's mean (% pixels) staining area was determined. ADAMTS-13, HMGB1, GR, Cu/Zn SOD and 8-OHdG immunohistochemical results were compared between groups using a one-way analysis of variance and Tukey's multiple comparison tests. *P* < 0.005 was considered statistically significant.

## Data Availability

All data generated or analyzed during this study are included in this published article.
